# Classical swine fever virus replicated poorly in cells from MxA transgenic pigs

**DOI:** 10.1186/s12917-016-0794-5

**Published:** 2016-08-17

**Authors:** Yicheng Zhao, Tiedong Wang, Li Yao, Bo Liu, Chunbo Teng, Hongsheng Ouyang

**Affiliations:** 1 College of Life Science, Northeast Forestry University, Harbin, China; 2 College of Animal Sciences, Jilin University, Changchun, China; 3 College of Veterinary Medicine, Inner Mongolia Agricultural University, Hohhot, China

**Keywords:** Classical swine fever virus, MxA, Nuclear transplantation, Transgenic pig

## Abstract

**Background:**

In addition to their value as livestock, pigs are susceptible to classical swine fever virus (CSFV) and can serve as reservoirs for CSFV, allowing it to develop into an epizootic. CSFV, a pestivirus of the Flaviviridae family, has a single-stranded RNA genome. Recent research has indicated that the human MxA protein inhibits the life cycles of certain RNA viruses, such as members of the Bunyaviridae family, the Flaviviridae family and others.

**Results:**

To produce pigs with antiviral protection against CSFV, transgenic pigs expressing human MxA were generated by nuclear transplantation. Cells from three MxA transgenic piglets were used to investigate in vitro antiviral activity of MxA aganist CSFV, and the results of in vitro indirect immunofluorescence assays, virus titration and real-time PCR indicated that the MxA transgenic pig has an antiviral capacity against CSFV.

**Conclusions:**

Transgene with human MxA on pigs is feasible. High levels of MxA expression do inhibit CSFV in vitro at early time points post-infection at 60-96dpi.

## Background

Human MxA has been shown to exhibit a wide antiviral capacity against small RNA viruses. This interferon-induced protein is one of the best-studied determinants of innate immunity to viral infection [1]. MxA belongs to the dynamin superfamily of large GTPases and has a C-terminal stretch of basic amino acids that constitutes a nuclear localization signal (NLS). This NLS mediates the nuclear accumulation of MxA, whereas the swine Mx1 protein is not located inside nucleus for lack of NLS. At present, the known MxA-sensitive viruses include members of the bunyaviruses, orthomyxoviruses, paramyxoviruses, rhabdoviruses and togaviruses, along with Hepatitis B virus [2–7].

Classical swine fever virus (CSFV) is a pestivirus of the Flaviviridae family [8] with an enveloped virion incorporating glycosylated membrane proteins. The CSFV genome has a single strand of positive-sense RNA [9] and a single open reading frame (ORF) that encodes a large protein that is cleaved and modified into several smaller proteins, including N^pro^, C, E0, E1, E2, P7, NS2-3, NS4A, NS4B, NS5A and NS5B. CSFV infects pigs and replicates predominantly in myeloid cells, causing classical swine fever (CSF), a severe disease that is characterized by fever, leukopenia and hemorrhage [9–11].

The expression of porcine Mx1 was absent from CSFV-infected pigs because of the Interferon-blocking effects of N^pro^ [12, 13]. Moreover, our earlier study concluded that highly expressed MxA inhibits CSFV in vitro [14]. As the inherently wide antiviral capacity of MxA, we sought to generate a transgenic pig model that would express a high level of MxA under the control of the EF1a promoter to protect swine against CSF. In this study, three MxA transgenic pigs were generated by Nuclear transplantation (NT), and we determined the levels at which CSFV replicated in cells from different tissues of these transgenic clone piglets.

## Methods

### DNA transfection and the establishment of stable cell lines

The plasmid pGKneotAloxp2MxA, which expresses human MxA in eukaryotic cells, was constructed in our previous study [14]. Porcine fetal fibroblasts (large white) cultured in Dulbecco’s modified Eagle’s medium (GIBCO) with 20 % fetal bovine serum (PAA) were seeded in a 60-mm dish prior for transfection. Then, the linearized plasmid was transfected into the porcine fetal fibroblasts using Fugene HD Transfection Reagent (Roche). After 14-36 h, the cells were split into 6-well dishes and cultured in selective medium containing 300 ng/mL of G418 antibiotic (Amresco) for 7-12 days. The surviving cell colonies were selected for further analysis.

### The generation of transgenic pigs by nuclear transplantation

Positive cell clones were used to construct transgenic pigs by Nuclear transplantation (NT). The details of the method of porcine NT used were described in our earlier work [15]. Briefly, oocytes were washed three times and then matured in maturation medium at 39 °C. In vitro-matured metaphase II oocytes were harvested by the removal of their polar bodies and the associated metaphase plates. An electrical pulse was used to fuse the donor cells and the oocytes. The reconstructed oocytes were electrically activated to initiate cell division and subsequent development. The embryos were cultured for 20 h following activation and were surgically transferred into the oviducts of surrogate pigs.

### DNA extraction, RNA extraction, PCR analysis and Integration site analysis

Genomic DNA from different tissues and cells was extracted and purified using a QIAamp DNA Mini Kit (QIAGEN). RNA was extracted with TRIzol reagent (Invitrogen) and purified using an RNeasy column (QIAGEN). The sites of transgene integration were assessed using high-efficiency thermal asymmetric interlaced PCR (hiTAIL-PCR) [16]. Primers specific to the transgenic vectors (pMl1, pMl2 and pMl3) were designed using Vector NTI 10 software (Invitrogen). The detailed method has been previously described [17].

All the primers used in our research and their sequences are listed below:

- MxAq1 GAGAGGAAACTGTAGGGGAG
- MxAq2 GGAAACATCTGTGAAAGCAA
- Neo1 TGAAGTAACTGCAGGACGAG
- Neo2 AATATCACGGGTAGCCCACG
- β-actin1 GAACCCCAAAGCCAACCGT
- β-actin2 CCTCGTAGATGGGCACCGT
- rFPprimer GGGATGATGGTCTCCTGATCAC
- rRPprimer CATCAAATTGGTAGGCCACTTTC
- Prob5B FAM-CCTTGCTCGCGAATTTCTCACCGATAMRA (TaqMan)
- Vs1 TCGCCCTTATTCCCTTTTTT
- Vs2 AGCAGCCGATTGTCTGTTGT
- pMl1 TCGGTGATGACGGTGAAAAC
- pMl2 CGGAGACGGTCACAGCTTGT
- pMl3 AGAGCAGATTGTACTGAGAG
- LAD1 ACGATGGACTCCAGAGCGGCCGC (G/C/A) N (G/C/A)NNNGGAA
- LAD2 ACGATGGACTCCAGAGCGGCCGC (G/C/T) N (G/C/T)NNNGGTT
- LAD3 ACGATGGACTCCAGAGCGGCCGC (G/C/A) (G/C/A) N(G/C/A) NNNCCAA
- LAD4 ACGATGGACTCCAGAGCGGCCGC (G/C/T) (G/A/T) N (G/C/T) NNNCGGT

### Viral infections, virus titration, the indirect immunofluorescence assay (IFA) and real-time PCR

These assays were performed according to Xu et al. [18]. CSFV (SM) from the Institute of Veterinary Drug Control, China, was used in this study. Positive anti-CSFV serum was kindly provided by Dr. CC Tu. To determine the viral titer, cells were infected with CSFV for 60 h, and samples of the medium were collected, serially diluted 10-fold from 10^-1^ to 10^-6^ and inoculated onto PK-15 cells. Each dilution was examined for viral proliferation using IFA. The TCID_50_ values were calculated using the Kärber method. For IFA, cells were infected with CSFV for 72 h, fixed in precooled 80 % acetone, and incubated with the positive anti-CSFV serum. A FITC-conjugated rabbit anti-pig IgG (Sigma) was used as the secondary antibody, and the cells were examined using an ECLIPSE TE2000-V (Nikon). Real-time PCR was performed with an iQ^tm^5 Multicolor Real-time PCR Detection System (Bio-Rad) using a QuantiTect™ Probe PCR Kit (QIAGEN). The special primers (rFPprimer, rRPprimer and Prob5B) for the CSFV NS5B gene have been previously described [14].

### Western blotting

The cells were washed with PBS and a hypotonic cell lysis buffer containing morpholinepropanesulfonic acid-buffered saline with Triton X-100 (15ul for 10^6^ cells). The cell extract was centrifuged at 12,000 x *g* to remove the cellular debris. Detailed methods regarding separating the sample by SDS-PAGE, transferring proteins to nitrocellulose membranes, and immunostaining will be described elsewhere. As a control, a monoclonal mouse anti-β-actin antibody (human/mouse, 1:1000, kangchen) was used. The MxA protein was immunostained with a mouse monoclonal anti-MxA (p78) antibody (human/mouse, 1:500 Novus Biologicals).

### Southern blotting

The sample was digested overnight with EcoRI at 36 °C (1.5ul for 1ug, NEB). Then, the gDNA was electrophoresed for 22 h in a 1 % agarose gel at 13 V and transferred to a Hybond þ nylon membrane (GE Healthcare) for 12 h. A DIG-labeled probe was generated using a PCR DIG Probe Synthesis Kit (Roche) with primers Vs1 and Vs2. Hybridization was performed overnight at 55 °C using a DIG Easy Hyb Kit (Roche).

## Results

**Fig. 1:**
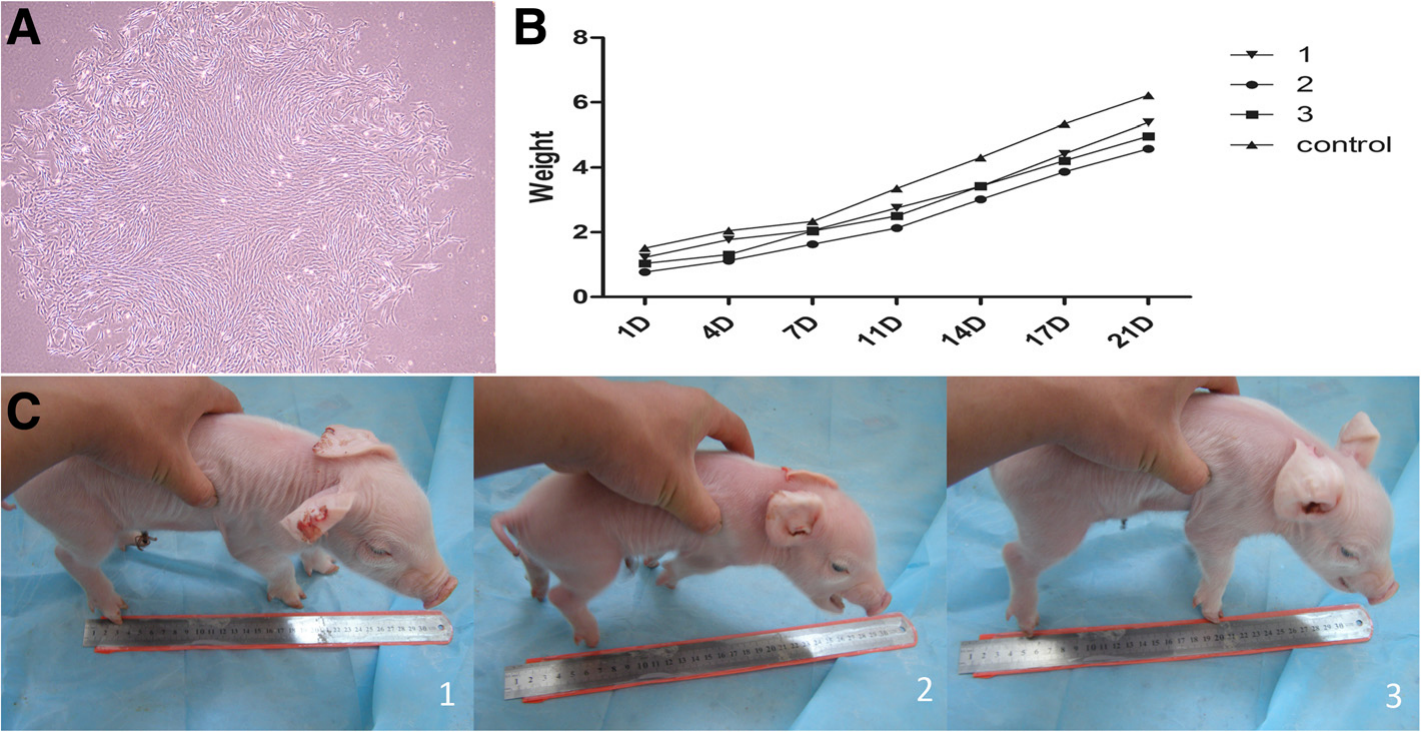
**a**. The number 34 clone was chosen for NT. Because porcine fetal fibroblasts are capable of a limited number of passages, the cells have a larger and longer shape in this picture. **b**. Three male transgenic piglets were obtained by natural delivery. **c**. The average birth weight was 1.01 kg, and the pigs displayed normal growth rates comparing a non-transgenic piglet born at the same day

To select supporting donor cells, several stable MxA-positive clones were constructed, infected with CSFV and analyzed (data not shown). The No. 34 clone (Fig. 1a) was chosen for NT, about 500 SCNT embryos were transferred into 3 surrogate mothers that exhibited natural estrus and three male transgenic piglets were obtained by natural delivery on day 119 post-implantation (Fig. 1b) and one piglet died before birth, the other three live transgenic piglets displayed no obvious weight differences comparing to a normal male piglet born at the same day (Fig. 1c).

**Fig. 2:**
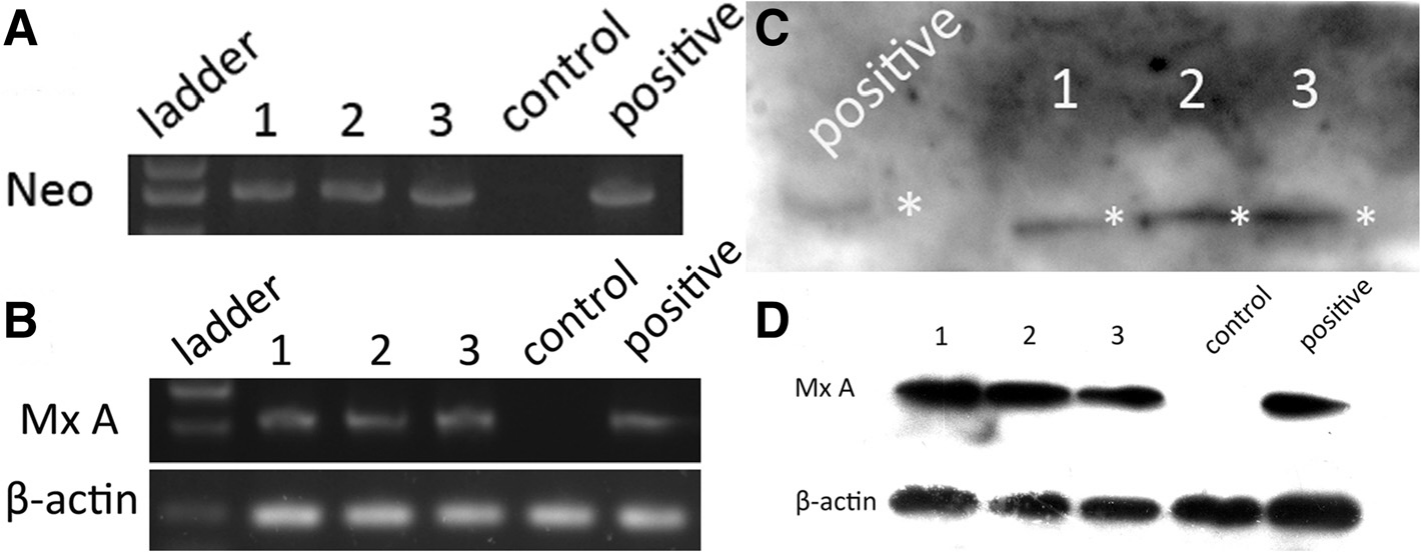
**a-b**. PCR data for the neomycin resistance gene (Neo), and RT-PCR data for MxA were performed to confirm the presence of the transgene; the No. 34 clone cells were chosen for the positive control, and normal porcine tails gDNA and blood RNA were chosen as negative control. **c**. Southern blotting was performed to detect the vector fragment in the genome; pGKneotAloxp2MxA was chosen as the positive control. **d**: Western blotting showed the expression of MxA in all three piglets; a cell strain stably expressing MxA was chosen for the positive control, and tissue from non-transgenic piglets was chosen as a negative control

The results of PCR detection of the neomycin resistance gene (Neo) from tail DNA and RT-PCR detection of MxA from blood RNA were both positive (Fig. 2a and b). The amplification of template genomic DNA with nested PCR primers yielded amplified product of the predicted size: 163 bp (outer set) and 107 bp (inner set). The PCR-amplified inner set product were sequenced. The sequence analysis revealed that the MxA fragment had randomly inserted into introns on chromosome 16 (, NW_003612429:30482-30588). Southern blotting targeting clone piglets tails’ DNA and Western blotting targeting porcine kidney cells were also performed to demonstrate the authenticity of the MxA expression in these transgenic piglets (Fig. 2c and d).

**Fig. 3:**
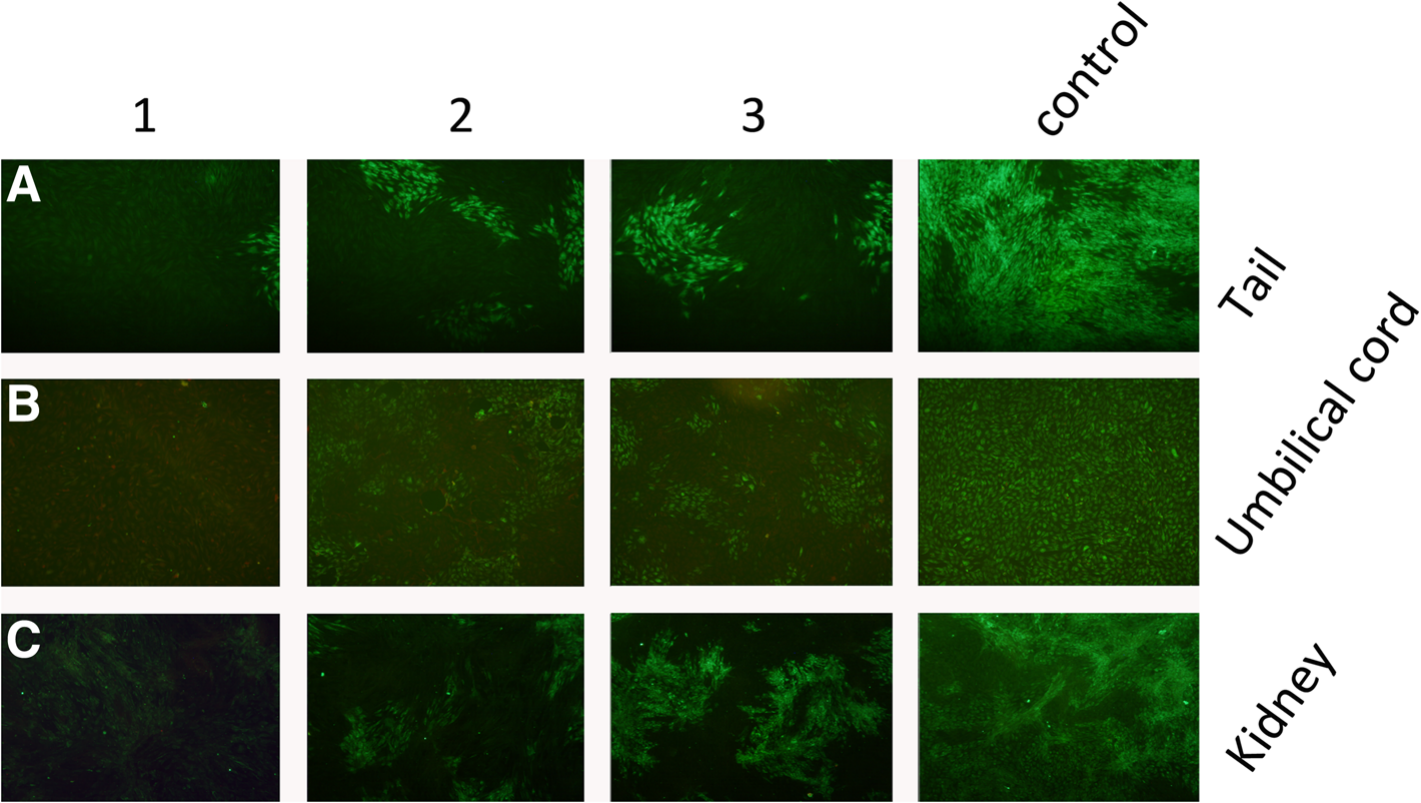
**a-c**. IFA demonstrated that the proliferation of CSFV was obviously inhibited in piglet cells, including tail, kidney and umbilical cord cells. Compared with the counterparts from non-transgenic piglet, only a few MxA clone pigs’ cells displayed green fluorescence. Moreover, we found that the kidney cells were more sensitive to CSFV than the other two cell types. Our results indicate that the transgenic piglets were effectively protected by MxA

**Fig. 4:**
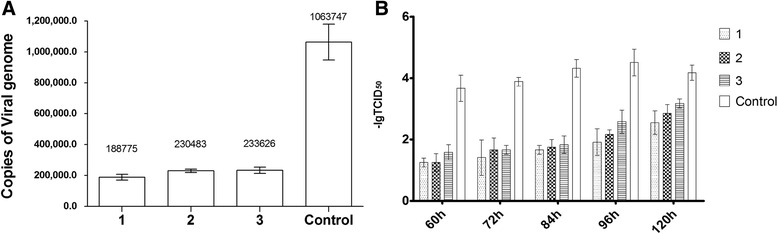
**a**. A reduction in the CSFV genome copy number at 96 h post-infection is shown; the copy numbers of CSFV are the means of three repeat experiments. The copy number (viral genomes per nanogram of total RNA) was 1.05 × 10^5^ copies/ng in the control cells, whereas there were 1.89 × 10^3^, 2.31 × 10^3^ and 2.34 × 10^3^ copies/ng in the MxA-positive cells. This result indicates the suppression of CSFV replication in MxA-positive groups. **b**. Inhibition of virus production in piglet cells. Cells were infected with 100 TCID_50_ of CSFV for 60 h, and the viral supernatant was diluted and inoculated onto PK-15 cells to assess the TCID_50_ values from 60 to 120 h. The -logTCID_50_ values represent the means of three repeat titrations after one time infection at each time point. The data indicate that MxA markedly inhibited infectious virus production for the first 3 days, but thereafter, the extent of the inhibition decreased

To detect CSFV replication, adherent cells from tails, kidneys and umbilical cords were isolated and cultured. The cells were infected with 100 TCID_50_ of CSFV for 72 h, and the infected cells were detected using IFA. Only a few MxA-expressing cells exhibited green fluorescence, indicating that most of the cells had been effectively protected by MxA and had resisted viral infection (Figs. 3a-c). To quantify the effect of MxA on viral replication, viral genome copy numbers from porcine tail cells were determined using real-time PCR at 96 hpi. The viral genome copy number per nanogram of total RNA was measured to be 1.05 × 10^5^ copies/ng of total viral RNA from control cells, whereas the MxA-positive cells contained 1.89 × 10^3^, 2.31 × 10^3^, and 2.34 × 10^3^ copies/ng, corresponding to 55-, 143- and 23-fold reductions, respectively (Fig. 4a). Moreover, the tail cells were infected with 100 TCID_50_ of CSFV for 60 h, and the virus-containing supernatant was diluted and used to inoculate PK-15 cells to determine the TCID_50_ values at 60, 72, 84, 96 and 120 h. As shown in Fig. 4b, the MxA protein markedly inhibited infectious virus production at the early time points(60-96hpi) comparing to normal porcine tail cells, however, at later times post-infection (120hpi), the degree of inhibition was reduced (Fig. 4b).

## Discussion

Transgenic pigs are of great value for commercial applications and could serve as models for human disease [19, 20]. Pigs are closer to humans in complexity, physiological function and genetics [15, 21]; therefore, we aimed to express human MxA, but not mouse Mx1, in pigs. Somatic cell nuclear transfer is a promising method of producing transgenic animals; however, this cloning strategy has a low success rate (<2 %) for bringing transferred pig embryos to term [22]. It has been well established that unknown mechanisms can affect the development, growth and survival of cloned animals [23]. Despite these potential constraints, we obtained three piglets (one additional piglet died before birth). These piglets did not display abnormal phenotypes in newborn bodyweight and auxodrome before weaning.

In this short report, the transgenes were characterized by PCR, integration site analysis, Southern blotting and Western blotting. To determine the antiviral capacity of MxA against CSFV, some basic in vitro experiments were performed. We used adherent cells from tails, kidneys and umbilical cords to evaluate the antiviral activities of the MxA transgene in the pigs. The results showed that CSFV replicated poorly in the cells of all three transgenic pigs, suggesting that MxA expression markedly inhibited viral growth, but at later time points post-infection(120hpi), the level of inhibition decreased.

## Conclusions

A conclusion from our study is that high levels of ectopic MxA expression in transgenic swine cells do inhibit CSFV in vitro at early time points post-infection, the inhibitory effects lasted for 96 h post the infection. We expect that this new transgenic model could facilitate animal production for antiviral research targeting RNA viruses.

## Abbreviations

CSFV: Classical swine fever virus
EF1a: Elongation factor 1 alpha
H5N1: Influenza A virus subtype H5N1
MXA: Myxovirus resistance gene A
NLS: Nuclear localization signal
NT: Nuclear transfer
ORF: Open reading frame
